# Serglycin promotes proliferation, migration, and invasion via the JAK/STAT signaling pathway in osteosarcoma

**DOI:** 10.18632/aging.203392

**Published:** 2021-09-07

**Authors:** Bin Lv, Guangyu Gao, Yuhong Guo, Zhiping Zhang, Renfeng Liu, Zhengzai Dai, Cheng Ju, Yiping Liang, Xiaofeng Tang, Min Tang, Xiao-Bin Lv

**Affiliations:** 1 Jiangxi Key Laboratory of Cancer Metastasis and Precision Treatment, The Third Affiliated Hospital of Nanchang University, Nanchang, China; 2 Department of Orthopedics, The Third Affiliated Hospital of Nanchang University, Nanchang, Jiangxi 330008, China; 3 Nanchang Key Laboratory of Orthopaedics, The Third Affiliated Hospital of Nanchang University, Nanchang, China; 4 Medical Department of Graduate School, Nanchang University, Nanchang, Jiangxi 330006, China; 5 Department of Oncology, The Second Affiliated Hospital of Soochow University, Suzhou 215004, Jiangsu, China; 6 Beijing Orthopaedics Hospital, Fourth Military Medical University, Xi'an, Shanxi, China; 7 Department of Radiotherapy and Oncology, Kunshan First People's Hospital Affiliated to Jiangsu University, Kunshan, Jiangsu Province, China

**Keywords:** SRGN, osteosarcoma, GEO, bioinformatics analysis, JAK/STAT

## Abstract

Background: Osteosarcoma (OS) is a common disease in the world, and its pathogenesis is still unclear. This study aims to identify the key genes that promote the proliferation, invasion, and metastasis of osteosarcoma cells.

Method: GSE124768 and GSE126209 were downloaded from the Gene Expression Omnibus (GEO) database. The gene ontology and enrichment pathway were analyzed by FunRich software. qPCR and Western blot were used to detect the gene expression. After gene knockdown, Transwell and wound healing assays were conducted on osteosarcoma cells to detect whether the genes were defined before enhancing the invasion of osteosarcoma.

Results: Totally, 341 mRNAs were found to be regulated differentially in osteosarcoma cells compared to osteoblasts. In addition, the expression level of Serglycin (SRGN) in osteosarcoma cells was higher than that in human osteoblasts. The invasion and proliferation ability of osteosarcoma cells with upregulated Serglycin was significantly increased, and on the contrary, decreased after Serglycin knockdown. Moreover, we preliminarily found that Serglycin may associate with the JAK/STAT signaling pathway.

Conclusions: By using microarray and bioinformatics analyses, differently expressed mRNAs were identified and a complete gene network was constructed. To our knowledge, we describe for the first time Serglycin as a potential biomarker.

## INTRODUCTION

Osteosarcoma (OS) represents the most common malignant bone tumors with high metastatic potential and poor prognosis in adolescents under the age of 20 [1]. It usually occurs in rapidly developing bones, such as the humerus, distal femur, or proximal tibia. Besides, it is a highly aggressive malignant tumor, often metastasizing to the lungs and other organs [2]. In recent years, with the application of adjuvant chemotherapy, the long-term survival rate of osteosarcoma has gradually increased from less than 20% to about 65%–70%, but some patients are still at risk of amputation or death [3]. Besides, the toxicity of some chemotherapeutic drugs is not avoided, and patient pain and burden also increase. The rapid development of high throughput array technology and improvement of data analysis techniques make it easy to find important genes associated with cancer [4]. Therefore, finding an important gene that affects the prognosis of osteosarcoma is still a hot research topic. The biological significance of the gene in the prognosis of osteosarcoma was examined.

Serglycin (SRGN) is a low molecular glycoprotein expressed in hematopoietic cells, endothelial cells, and macrophages. It is intracellular, secreted, and integrates into the extracellular matrix [5–7]. Previous researches have shown that the expression level of SRGN is up-regulated in many tumors, which contributes to tumor growth and metastasis, and is associated with poor prognosis [8, 9], including acute myeloid leukemia [10], breast cancer [11, 12], colorectal cancer [13], nasopharyngeal cancer [14, 15]. However, the role of SRGN in OS has not been considered. We do not yet know whether SRGN also promotes the occurrence and development of osteosarcoma cells. That is why we here search for genes that are relevant for the prognosis of osteosarcoma.

In this study, the differentially expressed genes in OS and normal osteoblasts (hFOB1.19) were analyzed by bioinformatics analysis. Quantitative real-time PCR, Western blot, and functional assays showed that SRGN was overexpressed in OS and promoted the invasion of OS. Finally, we briefly explored the role of SRGN in the JAK/STAT pathway. We found that the upregulation of SRGN expression in osteosarcoma cells promoted its proliferation, migration, and invasion.

## METHODS

### Microarray data

GEO database, the full name of gene expression omnibus, is a gene expression database created and maintained by National Biotechnology Information Center NCBI. It was constructed in 2000 and contains high-throughput gene expression data submitted by research institutions around the world. In our study, GSE124768 and GSE126209 were downloaded from the GEO database.

GSE124768, including 25 osteosarcoma samples and 25 control samples. Total: 50 samples. Gene expression profiling analysis of these samples was conducted on Illumina NextSeq 500 (Homo sapiens) (GPL18578). Dataset GSE126209 was processed by Illumina HiSeq 4000 (Homo sapiens) (GPL20301).

### DEMs analysis

GEO2R, an R-associated web application, was applied to filtrate DEMs between adenoma samples and adenocarcinoma samples. We also used R software to analyze two sets of data. The *p* < 0.05 and| log FC| ≥ 1 were considered as cutoff criterion.

### Functional and pathway enrichment analysis

GO functional analysis and KEGG pathway analysis were performed to predict the potential functions of the DEMs by using the Database for Annotation, Visualization, and Integrated Discovery (FunRich; http://www.funrich.org). Upregulated and downregulated DEMs were submitted to the FunRich online program. The top 10 items of the cellular component (CC), biological process (BP), and molecular function (MF) categories were then sorted and presented in the form of pie graphs. Cytoscape software was utilized to perform KEGG (Kyoto Encyclopedia of Genes and Genomes) enrichment analysis. ClueGO is a Cytoscape plug-in, which visualizes non-redundant biological terms for a large number of gene clusters in functional packet networks. By uploading target genes, the network graph according to kappa statistics was established.

### PPI network analysis

We performed the protein-protein network by utilizing String and Cytoscape software. It provides a large amount of information about the confirmed and predicted interactions between proteins. DEGs are then entered into the website. We think that the mRNA score > 0.7 is significant. Then, the PPI network is established by using Cytoscape.

### Cell culture

All the cell lines were kindly provided by Professor Kang (Sun yat-sen university). We cultured osteosarcoma cells (MG63, U2OS, 143B, U2R, they are common human osteosarcoma cell lines) and hFOB1.19 cells (human osteoblasts) in DMEM medium added 10% fetal bovine serum (FBS, Gibco) and cultured in a cell incubator with 5% CO_2_ and 85% relative humidity. All DMEM were purchased from Thermo Fisher Scientific (Shanghai, China).

### Transfection

SRGN plasmid primer was purchased from Wuhan GeneCreate Biological Engineering Co., Ltd, and pcDNA3.1 was used as the carrier. Short interfering RNA 1 (siRNA1), siRNA2, and the corresponding negative control (NC) were purchased from GenePharma Co. Ltd. (Suzhou, China). NC, siRNA1 and siRNA2 were separately transfected into 143B cells with Lipofectamine 2000 and Lipofectamine^®^ RNAiMAX purchased from Thermo Fisher Scientific (Shanghai, China). The procedure of this experiment strictly followed the instructions provided by Thermo Fisher Scientific. We collect the cells together after 48 hours of processing in preparation for the next experimental step needed. The sequences (11) used in our experiments are listed below: SRGN, 5′-atgatgcagaagctactcaaatg-3′ SRGN, 5′-ttataacataaaatcctcttctaat-3′ siRNA1, 5′-gaactacttccaggtgaatcc-3′ (sense), 5′-ggattcacctggaagtagttc-3′ (antisense); siRNA2, 5′-ggaacaggattaccaactagt-3′ (sense), 5′-actagttggtaatcctgttcc-3′ (antisense).

### Cell counting kit-8 assay

The CCK-8 kit was purchased from Shanghai Yeasen Biotechnology Co., Ltd., and used according to the manufacturer's instructions to detect the growth and proliferation of cells. Cells were inoculated in 96-well plates (3000 cells/well). The culture plates were precultured in an incubator for a while (37°C, 5% CO_2_). CCK-8 was added into 96 well plates at 0, 24, 48, 72 and 96 hours after transfection, and then cultured in incubator for 1 hour. Finally, we use Thermo Scientific Multiskan FC marker (Thermo Fisher Scientific, Shanghai, China) to measure the absorbance at 450 nm. Each assay was independently repeated three times. The growth curve was plotted by counting the number of living cells.

### Wound healing assay

To measure cell migration and repairability, we conducted wound healing assays. We used a 10 μ tip to generate uniform wounds in the central growth area of adherent cells cultured on a 12-well plate. The floating cells in the central part were washed with PBS, and the cells were cultured in a 37°C and 5% CO_2_ incubator for another 24 hours. Besides, we observed and filmed the migration process at the beginning and 36 hours after scratch. After that, we also measured the distance between the cells on both sides.

### Transwell assay

We performed a transwell assay to detect the invasion ability of cells. Millipore Transwell chambers were placed on a 24-well plate and osteosarcoma cells were digested with trypsin and washed with PBS. We added the cells treated with serum-free medium into the upper chamber and 1^*^10^5 cells were inoculated of each well, and added DMEM medium which contains 10% fetal bovine serum into the lower chamber. The difference between the invasion assay and the migration assay was that 50 ul diluted Matrigel (BD Biosciences, Franklin Lakes, NJ) was added to the upper chamber, the remaining steps are the same as the migration assay. The cells were cultured in a 37°C incubator for 22 hours in the migration experiment and 24 hours in the invasion experiment. The migrated or invaded cells were fixed, stained and counted at a specified time. We have conducted at least three experiments independently to verify the results. PBS, Trypsin, and serum-free medium were purchased from Thermo Fisher Scientific (Shanghai, China).

### Quantitative real-time PCR

We used TRIzol reagents (Invitrogen, Carlsbad, CA, USA) to extract total RNA from OS cell line according to the instructions provided by manufacturer. The total RNA was reverse-transcribed into cDNA using a PrimeScript™ RT kit with gDNA Eraser (TaKaRa, China). We also used SYBR Select Master Mix for CFX (Invitrogen) and the CFX Connect Real-Time PCR System (BioRad) to perform qRT -PCR. Amplification conditions have been listed below: 95°C for 15 seconds, and then 40 cycles of 95°C for 5 seconds, and 60°C for the 30s. Glyceraldehyde-3-phosphate dehydrogenase (GAPDH) was used as an endogenous control. Method 2^−ΔΔCt^ was used to analyze and process the record data. The qRT-PCR primer pairs were as follows [16]: SRGN forward, 5′-CGCTGCAATCCAGACAGTAA-3′, SRGN reverse, 5′-TCCCAGATCCTGATCCAGAG-3′, GAPDH forward, 5′-TGGTATCGTGGAAGGACTCATGAC-3′, GAPDH reverse, 5′-ATGCCAGTGAGCTTCCCGTTCAGC-3′.

### Western blot analysis

Wash the cells several times with PBS, then RIPA buffer containing protease inhibitors was added to the cell culture dish and placed on ice to lyse the cells for 30 minutes. Then centrifuge at 12000g at 4°C for 25 minutes. Then we measured the protein concentration with BCA protein analysis kit (Pierce, Rockford, IL, USA). Proteins were separated on 10% SDS-PAGE electrophoresed and transferred to PVDF membrane (polyvinylidene fluoride, Millipore, USA). The PVDF membrane was sealed for 1 hour with 5% skim milk at room temperature. Afterward, the membrane and the corresponding antibody were incubated at 4°C overnight (The order number of SRGN is NBP1-80886). The next day, the membrane was washed with PBST three times and incubated with corresponding secondary antibody for 1 hour, then washed three times with PBST and developed with ECL kit. β-actin is the internal control.

### Statistical methods

The data were expressed as the mean ± standard error of the mean. R software and GraphPadPrism6 package were utilized to conduct data analysis. The student’s *t*-test was utilized to compare the differences between two dependent groups. All experiments were performed in triplicate.

### Availability of data and materials

The datasets used and/or analyzed during the current study are available from the corresponding author on reasonable request.

## RESULTS

### Data preprocessing and differential expression analysis

**Figure 1:**
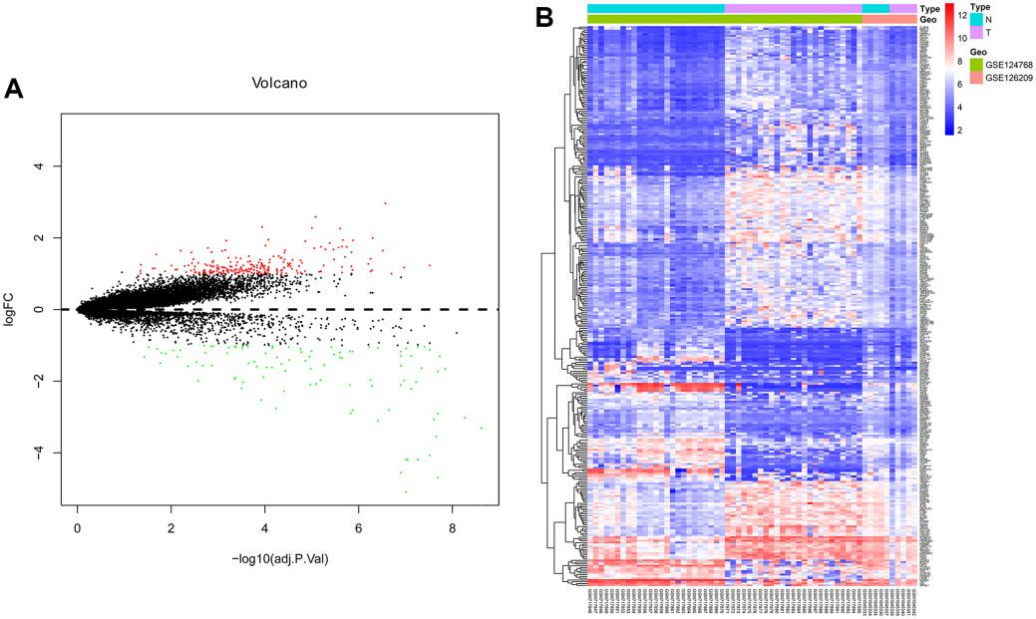
**Differentially expressed genes analyzed by R software.** (**A**) Volcano plot and (**B**) Heat map of differentially expressed genes. A: Red dots indicate up-regulated DEGs, green dots indicate down-regulated DEGs, black dots indicate non-differentially expressed DEGs. B: Red dots indicate up-regulated DEGs, blue dots indicate down-regulated DEGs, white dots indicate non-differentially expressed genes.

To identify significantly DEMs and DEGs taken part in the development of OS, R software was used to research the gene expression profiles from the GSE124768 and GSE126209. Based on the cut-off criteria (adj.P.Val<0.05 and |log2FC|≥1), 352 differentially expressed mRNAs were identified, including 207 upregulated mRNAs and 145 downregulated mRNAs. upregulated (red) and downregulated (blue) mRNAs between OS cells and healthy donor cells were separately shown on the volcano plot and heatmap respectively (Figure 1).

### KEGG pathway analysis and GO enrichment analysis

**Figure 2:**
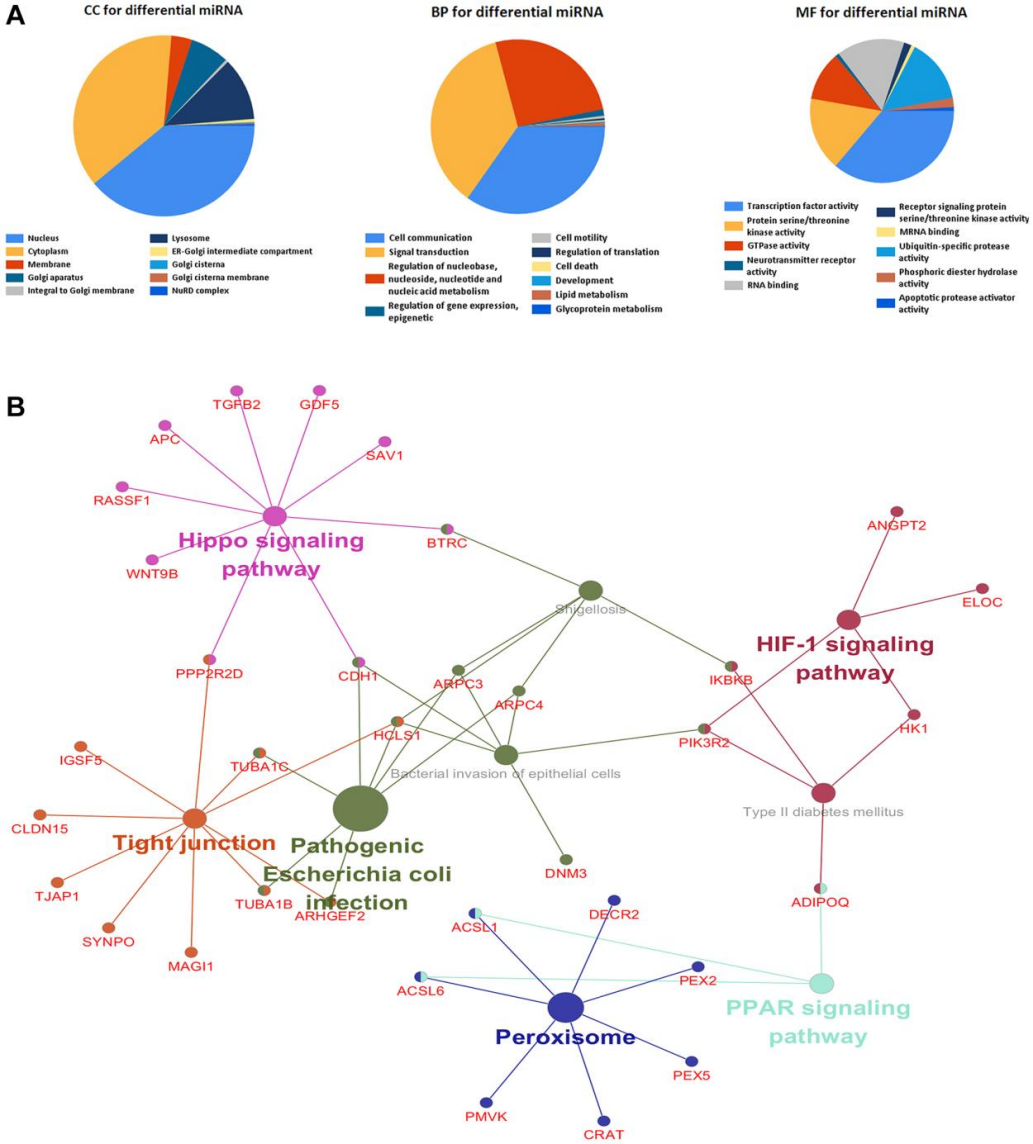
**Differentially expressed genes were analyzed by gene ontology and KEGG enrichment.** (**A**) The top 10 of biological process, cellular component, and molecular function of identified genes. (**B**) KEGG pathway enriched by selected genes.

mRNAs were uploaded into FunRich to conduct GO analysis. The result indicated that differentially expressed mRNAs were most enriched in the cytoplasm, nucleoside, mRNA binding, cell communication, signal transduction and cell communication, and transcription factor activity (Figure 2A). KEGG pathway analysis showed that these potential target genes were mainly enriched in 6 pathways including the PPAR signaling pathway, Peroxisome, Hippo signaling pathway, Tight junction, HIF-1 signaling pathway, and Pathogenic *Escherichia coli* infection (Figure 2B). According to previous studies, their Functional category, cell location, and signaling pathways are associated with the development of diverse malignant tumors.

### Construction of a PPI network

**Figure 3:**
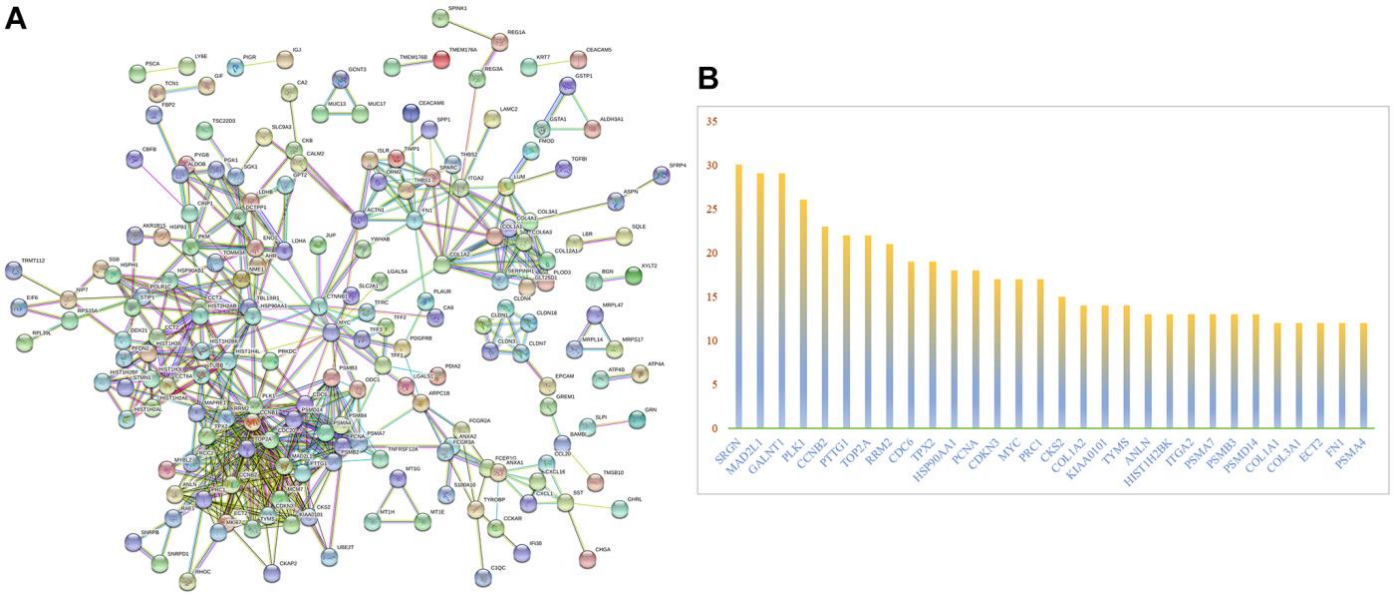
**PPI network analysis of differentially expressed mRNAs.** (**A**) PPI network analysis. (**B**) The frequency with which each core gene appears in the network diagram.

Protein-protein interaction network (PPI) is a network composed of proteins through interaction, which is involved in biological signal transmission, gene expression regulation, energy and material metabolism, cell cycle regulation and other aspects of life processes. Systematic research of protein-protein interactions in biological systems is vital to understand the working principle of proteins in biological systems, understand the reaction mechanism of biological signals and energy metabolism under special physiological conditions, and understand the functional relationship between proteins. 352 genes were entered into the String website to get a PPI network (Figure 3A). In the PPI network, we studied the number of nodes corresponding to each protein including SRGN, MAD2L1, GLANT1, PLK1, PTTG1, CCNB2, and TOP2A. In addition, we draw the histogram of the frequency of the selected gene nodes (Figure 3B). SRGN has the highest number of nodes, so we chose it as the object of further study.

### SRGN was upregulated in OS cells

**Figure 4:**
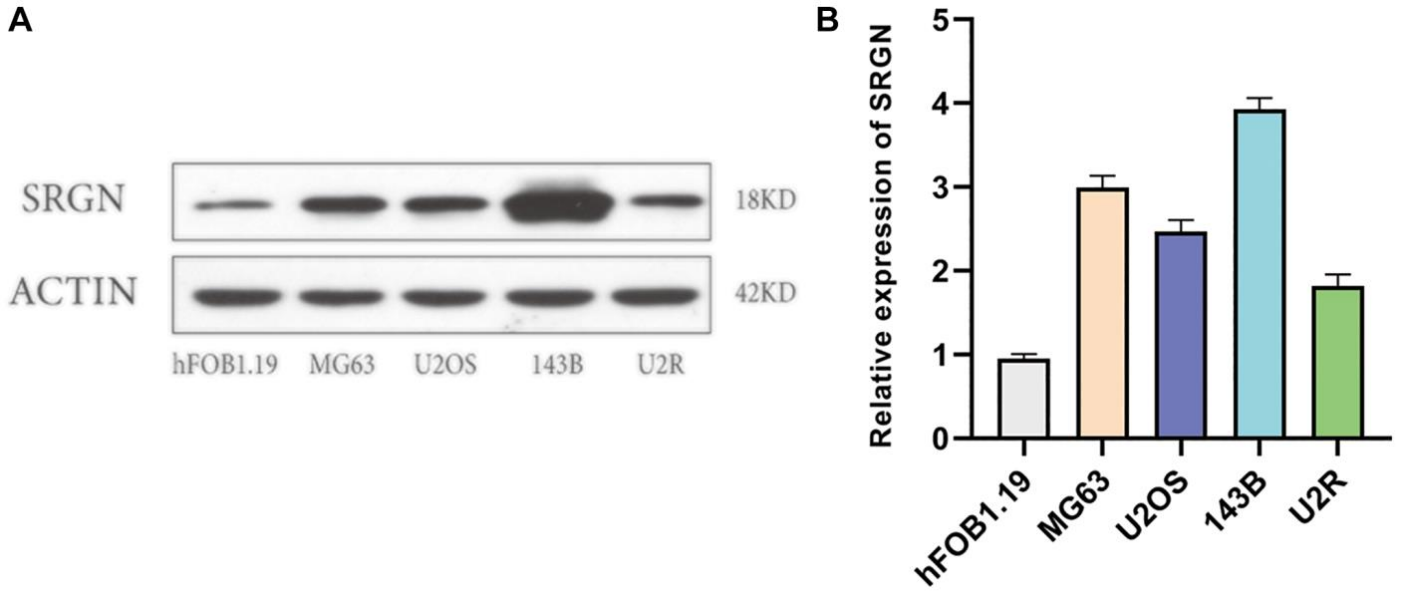
**SRGN expression was upregulated in osteosarcoma cells.** (**A**) Expression of SRGN by western blot in hFOB1.19 and osteosarcoma cells. (**B**) The expression level of SRGN in osteosarcoma cells was detected by quantitative polymerase chain reaction (qRT-PCR). Data are presented as mean ± SD. ^*^P < 0.05.

In previous studies, we have found that SRGN expression is upregulated in many tumors, such as NSCLC [17], BC [11, 12], and CRC [13]. Unfortunately, whether SRGN expression is upregulated in osteosarcoma cells as in other tumors has not been reported. To explore the expression of SRGN in OS cells, qRT-PCR and Western-blot assays were used to analyze the expression levels of SRGN at mRNA and protein levels. In the cell line shown in Figure 4, hFOB 1.19 is a human osteoblast, which serves as a control group, other cells such as 143B, U2OS, MG63, and U2R are human osteosarcoma cell lines. The results showed that SRGN expression was the highest in 143B cells at both mRNA level and protein level, while the expression of SRGN in other OS cell lines was lower than that in 143B cells (Figure 4), and the expression of SRGN in all OS cell lines was higher than that in normal human osteoblasts (hFOB1.19).

### SRGN promotes the proliferation of 143B cells

**Figure 5:**
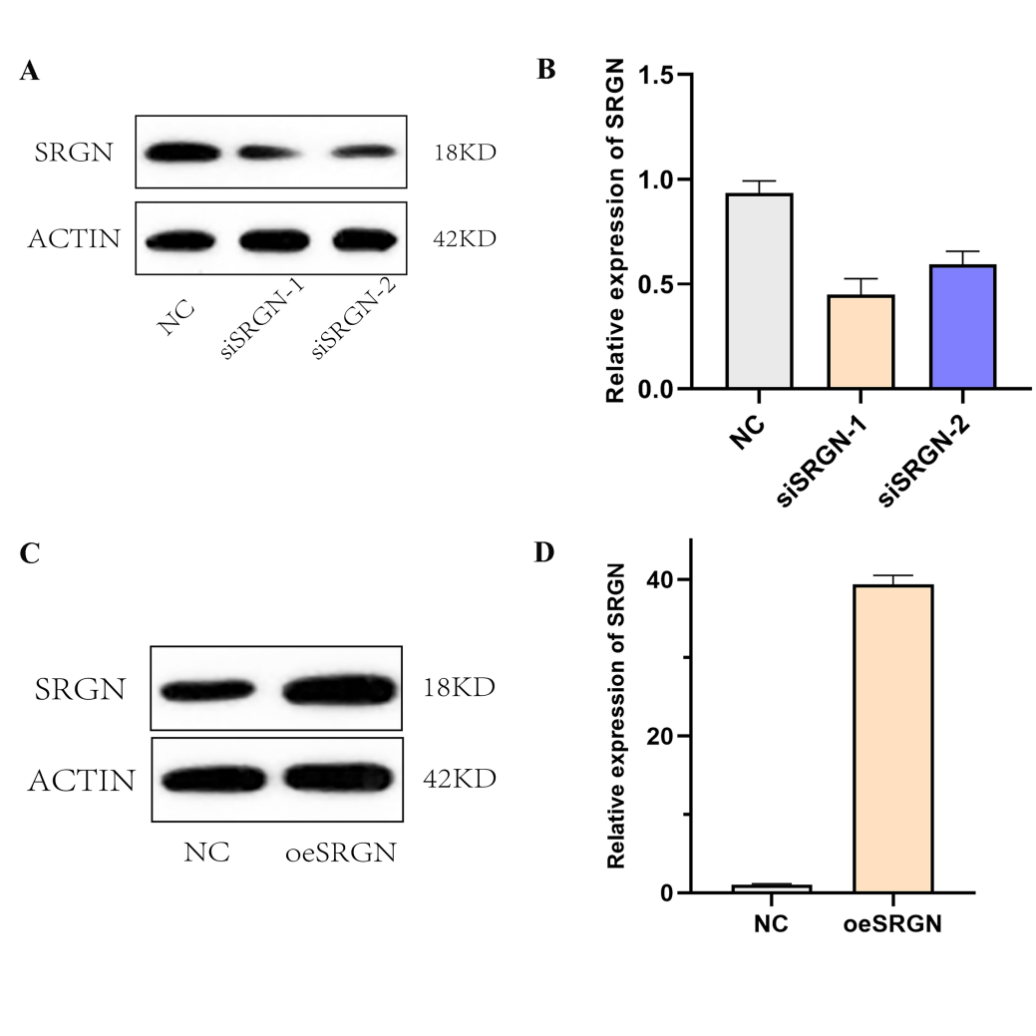
**Overexpression and knockdown efficiency of SRGN.** (**A**) and (**B**). The interference efficiency of siRNA1 and siRNA2 against 143B was detected by Western blot and qRT-PCR, respectively. (**C**) and (**D**). The overexpression of SRGN at the protein level and mRNA level.

**Figure 6:**
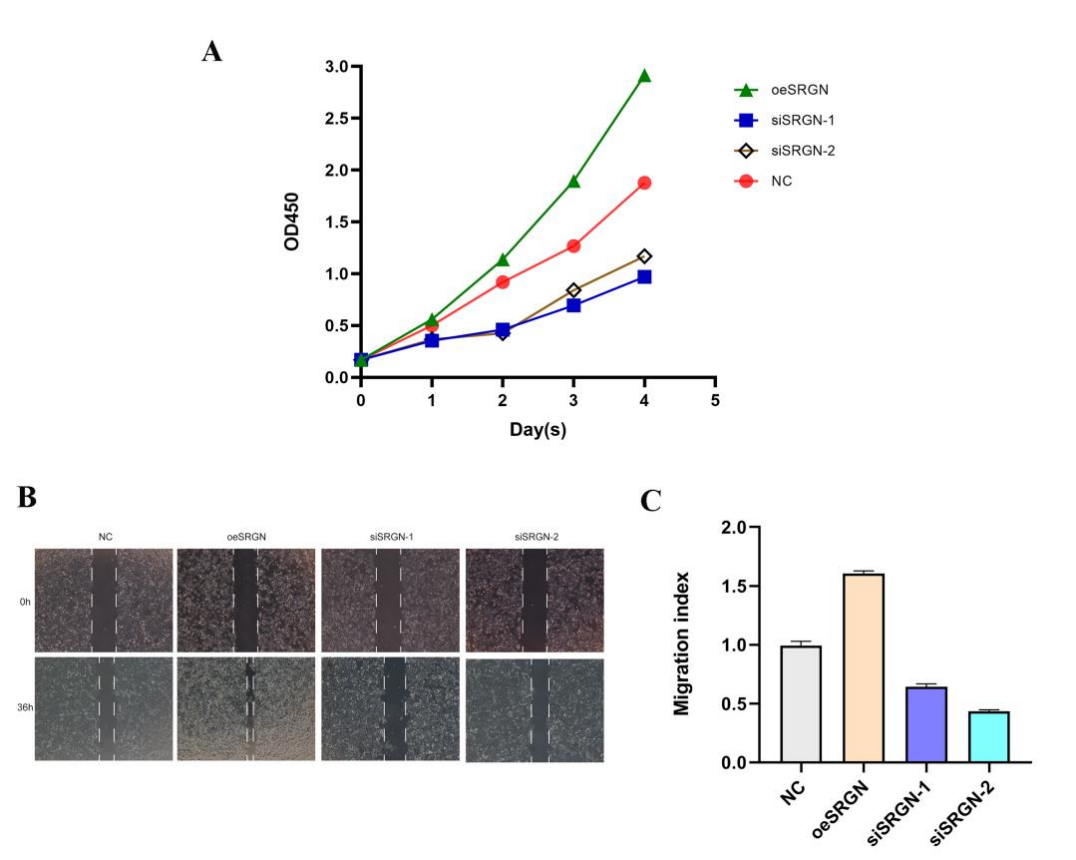
**SRGN knockdown inhibited the proliferation of OS cells.** (**A**) The effect of upregulation and silencing of SRGN on proliferation in 143B cells was detected by CCK8. (**B**) and (**C**) The migration ability of 143B cells was investigated.

Based on the previous results, we selected 143B cells for next assays. We transfected SRGN plasmid, siSRGN-1, and siSRGN-2 into 143B cells, respectively. Figure 5A and 5B showed that the expression of SRGN in siRNA transfected cells was significantly lower than that in NC cells, which was the same at different expression levels. The results indicated that these siRNAs can reduce the expression of SRGN in 143B cells. Similarly, we also detected the overexpression efficiency of SRGN in 143B cells by Western blot and qRT-PCR assays, as shown in Figure 5C and 5D. Previous researches have shown that the high expression of SRGN in other tumors promotes its tumor aggressiveness and proliferation. To investigate whether SRGN promoted or reduced the proliferation of 143B cells, we conducted the CCK-8 assay, as shown in Figure 6A, overexpression of SRGN (oeSRGN) could enhance the proliferation of 143B cells, while od value of the two knockdown groups was lower than that of the control group. In wound healing assays (Figure 6B and 6C), The results showed that the healing speed of SRGN overexpressed cells was the fastest at 36 h, while the healing speed of siSRGN-1 and siSRGN-2 transfected cells was significantly slower than that of NC cells. Therefore, SRGN can promote the proliferation of 143B cells.

### The upregulation of SRGN increased the aggressiveness while knockdown decreased it

**Figure 7:**
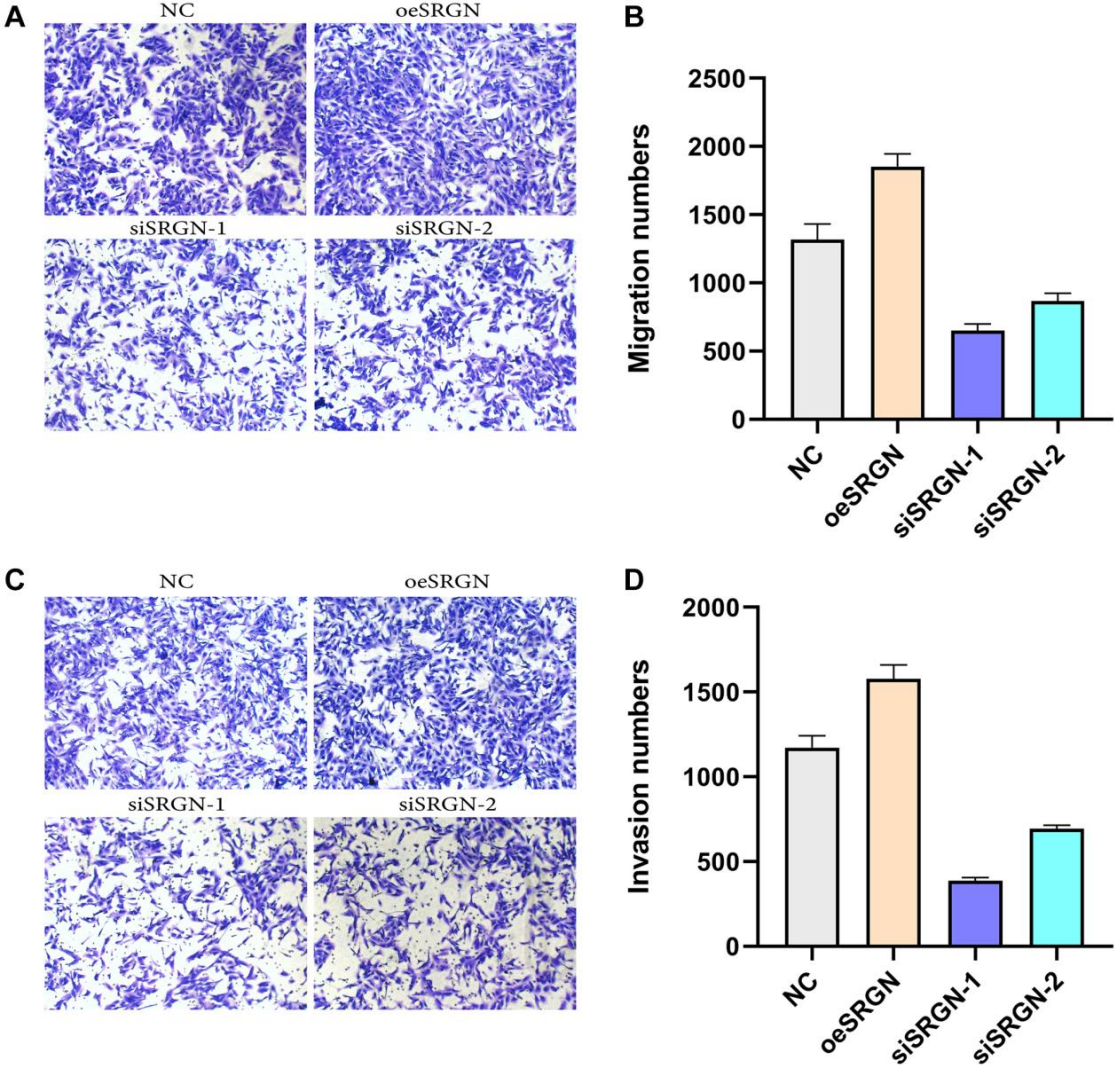
**SRGN affects the migration and invasion of 143B cells.** Transwell assays were used to detect the migration and invasion ability of 143B cells.

We transfected SRGN plasmid, siSRGN-1, and siSRGN-2 into 143B cells respectively, and carried out scratch, migration, and invasion assays. In the Transwell assays, from Figure 7, we found that compared with NC cells, the migration and invasion ability of SRGN overexpressed cells was enhanced, while the invasion ability of siSRGN-1 and siSRGN-2 transfected cells was significantly reduced. These results showed that overexpression of SRGN could enhance the aggressiveness and repairability of 143B cells, while the aggressiveness of 143B cells was significantly lower than that of NC cells after SRGN was knocked down.

### SRGN affects the JAK/STAT signaling pathway in OS cells

**Figure 8:**
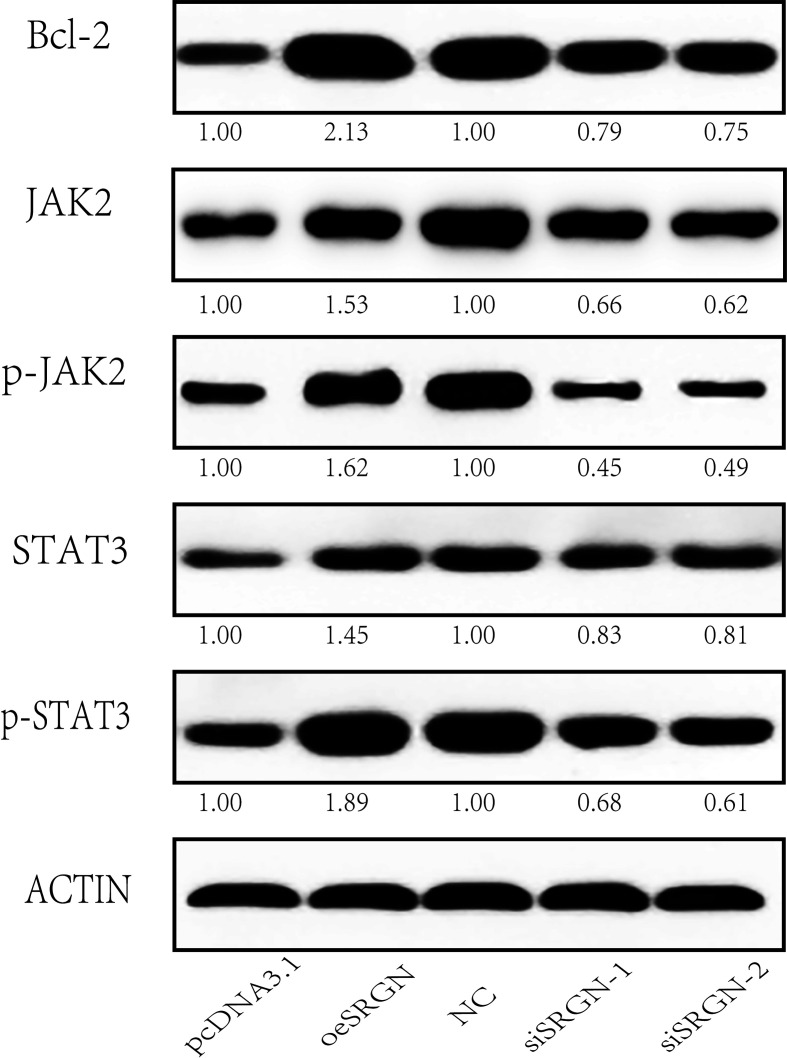
**SRGN affects the JAK/STAT signaling pathway in 143B cells.** Western blot analysis of the expression of Bcl-2, JAK2, p-JAK2, STAT3, p-STAT3, and ACTIN. pcDNA3.1, means MOCK transfection with pcDNA3.1, and it was used as the empty control of the overexpression group; NC, negative control used as the control for siRNA experiments; * We set the quantized value of pcDNA3.1 and NC to 1.00. The other quantized values were compared with their respective experimental groups.

Studies have shown that osteosarcoma progression is associated with JAK/STAT signaling dysregulation [18, 19]. We wondered whether SRGN also affects the JAK/STAT pathway in osteosarcoma cells. Thus, we overexpressed SRGN in 143B cells and transfected two siRNA into 143B cells, respectively, to research the effect of SRGN on the JAK/STAT pathway. The result is shown in Figure 8, compared with the control group, overexpression of SRGN reduced c-MYC, JAK2, and STAT3 expression levels, while silencing SRGN increased the phosphorylation of JAK2 and STAT3. In summary, SRGN may have influenced the JAK/STAT signaling pathway in 143B cells.

## DISCUSSION

In recent years, patients with osteosarcoma after treatment have a 5-year survival rate of 65–70%, but the survival rate of metastatic disease is lower, less than 20% [20]. In the case of poor treatment of metastatic osteosarcoma, we can study at the molecular level and find genes that progress the incursion, increment, and transfer of osteosarcoma cells. By inhibiting the expression of genes we selected, we can determine whether it can affect the activity of the bone tumor cell to develop new molecular targeting drugs.

In our study, we retrieve the gene expression data of GSE124768 and GSE126209 from the GEO. We identified 352 gene expression patterns related to cancer, indicating that these 352 genes play an important role in promoting the development of osteosarcoma. To learn more about the functional role of these mRNAs in osteosarcoma cells, FunRich was utilized for further research. The results showed that these genes were mainly associated with the cytoplasm, nucleoside, transcription factor activity, and RNA binding. This is consistent with current research that transcription factor activity and cell proliferation regulator function defects are the main reasons for cancer development [21, 22]. In cancer cells, ion transport is essentially different from that in normal cells [23]. KEGG pathway analysis concluded that these identified mRNAs were mainly enriched in 6 pathways including the PPAR signaling pathway, Peroxisome, Hippo signaling pathway, Tight junction, HIF-1 signaling pathway, and Pathogenic *Escherichia coli* infection. Several studies on the PPAR signaling pathway have shown that it participates in various important cellular processes [24, 25]. As for the HIF-1 signaling pathway, a previous study provides evidence that the inhibitor has been utilized as a treatment for Parkinson’s disease [26]. Also, recent studies indicated that the Hippo signaling pathway is involved in prognosis of metastatic melanoma, invasion, metastasis, and may be potential therapeutic targets [27]. However, the regulation mechanism of these pathways in osteosarcoma has not been researched yet.

In this research, we identified many different mRNAs by using bioinformatics analysis. According to constructing the PPI network, the number of nodes of SRGN was found to be the most. In previous studies, the number of SRGN was found to be highly expressed in multiple tumors and promoted the invasion of tumor cells. Therefore, we selected and detected whether there was a difference in the expression level of SRGN in normal human osteoblasts and osteosarcoma cell lines. From the above Western-blot and qRT-PCR results, it was found that the expression level of SRGN in osteosarcoma was indeed much more than that in human osteoblasts (hFOB1.19). Therefore, we chose to silence SRGN or express SRGN in 143B cells, and conducted CCK8 assay, Transwell assay, and wound healing assay for the treated cells. The results indicated that SRGN knockdown did reduce the proliferation of 143B cells, and overexpression of SRGN increased the ability of invasion and healing. We then found that SRGN may enhance the proliferation of 143B cells by acting on the JAK/STAT signaling pathway. Many important cellular processes in the human body have participation in JAK/STAT signaling pathways, including cell proliferation, differentiation, apoptosis and immunomodulation. In our study, we found that knockdown SRGN decreased JAK2 and STAT3 expression levels, while overexpression of SRGN increased their expression levels. As we all know, JAK / STAT signaling pathway is a key factor in the occurrence of many kinds of malignant tumors. From Figure 8, we can find that overexpression of SRGN increases the phosphorylation level of JAK2 and STAT3 and activates the anti-apoptotic gene Bcl-2. However, knockdown is contrary to overexpression. Overexpression and continuous activation of STAT3 can upregulate the anti-apoptotic protein (Bcl-2) to inhibit the apoptosis of tumor cells and prolong the cell life cycle. In conclusion, we can infer that SRGN plays a role in promoting tumorigenesis and aggressiveness in osteosarcoma.

At present, with the development of cancer treatment, individual difference therapy has been paid more and more attention. Therefore, it is important to discover novel therapeutic targets and methods for cancer patients. Our research for the diagnosis and prognosis of osteosarcoma provides a potential therapeutic target, but the generation of osteosarcoma has a variety of reasons, the onset process also involves many factors, and our research still not perfect, such as how SRGN affects JAK/STAT signal pathway, and its possible mechanism of SRGN is unclear and so on, all this needs further research. Because all of our data are obtained from the GEO and TCGA database through R software, further data analysis and basic experiments are needed to verify.

## CONCLUSIONS

We studied the influence of SRGN on the proliferation and invasion of OS cells. Plenty of differentially expressed genes were identified between normal samples and tumor samples by utilizing bioinformatics methods. Also, we examined the expression level and invasiveness of this gene in osteosarcoma and found that overexpression of SRGN enhanced the invasiveness of osteosarcoma, which may be related to the malignant prognosis of osteosarcoma. These results may give a potential therapeutic target for future clinical treatment.
